# Standardizing Umbilical Cord Mesenchymal Stromal Cells for Translation to Clinical Use: Selection of GMP-Compliant Medium and a Simplified Isolation Method

**DOI:** 10.1155/2016/6810980

**Published:** 2016-02-04

**Authors:** J. Robert Smith, Kyle Pfeifer, Florian Petry, Natalie Powell, Jennifer Delzeit, Mark L. Weiss

**Affiliations:** ^1^The Midwest Institute for Comparative Stem Cell Biotechnology, Department of Anatomy and Physiology, Kansas State University, Manhattan, KS 66506, USA; ^2^Institute of Bioprocess Engineering and Pharmaceutical Technology, University of Applied Sciences Mittelhessen, 35390 Giessen, Germany

## Abstract

Umbilical cord derived mesenchymal stromal cells (UC-MSCs) are a focus for clinical translation but standardized methods for isolation and expansion are lacking. Previously we published isolation and expansion methods for UC-MSCs which presented challenges when considering good manufacturing practices (GMP) for clinical translation. Here, a new and more standardized method for isolation and expansion of UC-MSCs is described. The new method eliminates dissection of blood vessels and uses a closed-vessel dissociation following enzymatic digestion which reduces contamination risk and manipulation time. The new method produced >10 times more cells per cm of UC than our previous method. When biographical variables were compared, more UC-MSCs per gram were isolated after vaginal birth compared to Caesarian-section births, an unexpected result. UC-MSCs were expanded in medium enriched with 2%, 5%, or 10% pooled human platelet lysate (HPL) eliminating the xenogeneic serum components. When the HPL concentrations were compared, media supplemented with 10% HPL had the highest growth rate, smallest cells, and the most viable cells at passage. UC-MSCs grown in 10% HPL had surface marker expression typical of MSCs, high colony forming efficiency, and could undergo trilineage differentiation. The new protocol standardizes manufacturing of UC-MSCs and enables clinical translation.

## 1. Introduction

The minimal criteria for defining mesenchymal stromal cells (MSCs) were provided by the International Society of Cellular Therapy (ISCT) MSC working group in 2006 and updated in 2013 with guidelines for characterization of MSC immune properties [1–4]. The physiological properties of MSCs suggest a potential to treat diseases such as graft versus host disease (GVHD) and Crohn's [5–7]. In addition, there are more than 500 clinical trials testing the safety and efficacy of MSCs listed on ClinicalTrial.GOV [8].

In 2014, about 53% of the MSC clinical trials worldwide used bone marrow-derived MSCs (BM-MSCs) [9]. BM-MSCs may be used as an autologous cellular product, which is a distinct advantage over allogeneic MSC products. However, the collection of BM is a painful, invasive procedure, when compared to MSCs from umbilical cord stroma (UC-MSCs) which is collected painlessly from tissues that are discarded after birth. Furthermore, adult BM-MSCs have a lower expansion potential, lower immunosuppression capability when cocultured with activated T-cells, and perhaps a more restricted differentiation potential than UC-MSCs [10–15].

UC-MSCs have advantages over BM-MSCs when considered as an allogeneic MSC source. These advantages include a virtually limitless supply of starting material which is available for producing tissue banks for use as an allogeneic matched product, much like umbilical cord blood banks, the collection of umbilical cords is painless, and the cord donors are of a consistent, young age.* In vitro*, UC-MSCs have high proliferation potential, broad differentiation potential, and improved immune modulation properties [11, 16–18]. For these reasons, the therapeutic potential of UC-MSCs bears testing, and 25 clinical trials worldwide were using UC-MSCs as of 2014 [9].

There are “challenges” to produce MSCs meeting requirements for clinical application [2, 19]. This has led to speculation that MSC manufacturing capacity may not keep pace with the number of MSC clinical studies [19, 20]. These challenges include the lack of a standardized method for isolating, expanding, and validating MSCs. For example, several methods to isolate UC-MSCs from umbilical cord stroma have been described [21] that include the tissue explant method [22, 23], mechanical dissociation of the cord stroma followed by enzymatic digestion [11, 23, 24], isolation of MSCs from the entire umbilical cord including the blood vessels [21, 22], enzymatic digestion of the tissue immediately surrounding the umbilical blood vessels [25], or mincing and enzymatic digestion of the stroma (Wharton's Jelly) without the blood vessels [24]. Several of these methods require dissection of the umbilical vessels. This dissection step increases processing time and the risk of contamination. For this reason, the goal here was to develop an isolation method which would decrease contamination risk and isolation time and increase the yield of MSCs. In reviewing our MSC expansion protocol, we determined that our medium formulation contained many ingredients and that this created a barrier for clinical manufacturing [24]. Therefore, our second goal was to identify a simplified medium that would provide for robust expansion of MSCs, be xenogen-free, and be suitable for clinical manufacturing.

## 2. Materials and Methods

### 2.1. Umbilical Cords

This research was deemed nonhuman subjects research by the institutional review board of Kansas State University since discarded, anonymous human tissue with all identifying linkages broken was used (IRB #5189). Tissue processing was performed inside a biological safety cabinet (BSC) in a BSL2 laboratory using universal precautions per Occupational Safety and Health Administration (OHSA) recommended blood borne pathogens containment described in 29 CFR. 1910.1030.

In a pilot study whose data is not presented here, 8 umbilical cords were used to identify optimization variables. In the work reported here, 24 umbilical cords (11 females and 13 males) were used; umbilical cords from vaginal births or Caesarean-section births were used. The umbilical cords were stored in sterile tissue sample container in saline solution at 4°C until use. In pilot work not presented here, umbilical cords were stored for up to 5 days prior to processing to extract MSCs; however, no parametric testing was performed to determine whether storage alters the quality of the product. Here, isolations procedures were performed within 4 days after birth. To randomize the treatment effects, we performed no prescreening and randomly assigned cord samples (biological replicates) to each experimental variable.

### 2.2. Isolation Optimization Strategy

Here our previously described protocol [24] was optimized to decrease contamination risk, increase yield, and improve GMP compatibility. For each umbilical cord (the biological unit), eight randomly selected 1 cm length samples were used to test the effect on the experimental variables identified in the pilot work. Two to four optimization variables were evaluated per cord using technical duplicates and the results were averaged for each experimental variable per biological unit for comparisons. First, we tested mechanical disruption of the tissue using a Miltenyi GentleMACS Dissociator (#130-093-235) using preprogrammed settings A, B, C, D, and E (which corresponds to weakest to strongest dissociation). Next, tissue dissociation conducted before or after enzymatic digestion was tested. Then, the effect of mincing the tissue samples was compared to tissue dissociation using the GentleMACS Dissociator. Next, the effect of filtering using 100 *μ*m cell strainers (Fisherbrand #22-363-549) and 60 *μ*m Steriflip tubes (Millipore #SCNY00060) was tested. Lastly, the concentration of enzyme was varied to determine the effect on yield. The technical duplicates or triplicates were averaged for each variable per cord sample. Each procedural optimization variable was evaluated using at least three different cord replicates. Decision making strategy was designed-based using process yield (more live cells) or increasing process efficiency (reducing number of processing steps, reducing time, or reducing contamination risk).

### 2.3. Final (Optimized) Isolation Method

**Figure 1:**
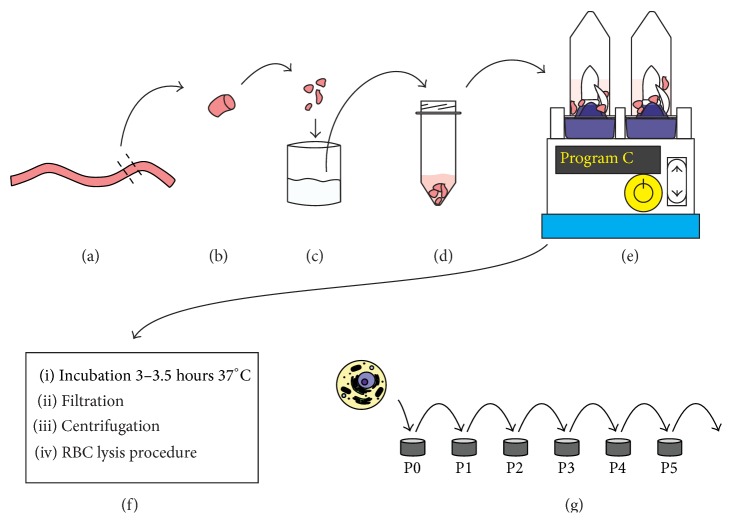
A schematic of the optimized isolation method. The major steps: (a) umbilical cord selected. (b) 1 cm section prior to cutting into 4 equal pieces. (c) Cord pieces rinsed in DPBS. (d) The cord pieces inside a C-tube immersed in enzyme solution. (e) Dissociation with C-tubes and Miltenyi Dissociator. (f) Steps following dissociation prior to plating the isolated cells. (g) The isolated cell initial plating at P0 and subsequent expansion over multiple passages.

A schematic of the revised method is shown in Figure 1. Umbilical cords were rinsed to remove surface blood using 37°C DPBS which had 1% Antibiotic-Antimycotic (Dulbecco's Phosphate Buffered Saline, Life Technologies #14190-250; Antibiotic-Antimycotic, Life Technologies #15240-062). The cords were then treated with 0.5% Betadine (Dynarex, Providone Iodine Solution, #1416) in DPBS for 5 minutes at room temperature. Inside the biological safety cabinet (BSC), the cord was cut into 1 cm lengths and rinsed repeatedly with 3 volumes of DPBS until no further surface blood could be seen. Each 1 cm length of tissue was cut into four equal size pieces and placed into a Miltenyi Biotech Dissociator C-Tube (Miltenyi #130-096-334). The tissue weight was calculated by subtracting the tare weight of the C-tube and 9 mL of enzyme solution was added. The C-tubes were placed into a Miltenyi Dissociator, processed using program C and incubated for 3–3.5 hours at 37°C with constant 12 rpm rotation. Following the 3–3.5-hour incubation, the tissues were dissociated using program B and filtered through 60 *μ*m Steriflip filter (Millipore #SCNY00060) to remove tissue debris. The cells were pelleted by centrifugation at 200 ×g for 5 minutes at room temperature and the supernatant was discarded. The cells were suspended in 0.5 mL of growth media and 0.5 mL RBC lysing solution (Sigma's RBC lysis solution, #R7757-100ML) was added to remove red blood cell contamination. The cells were mixed gently for one minute followed by addition of 8 mL of DPBS. Cells were centrifuged at 200 ×g for 5 minutes at room temperature and the supernatant was discarded. The cells were suspended in 1 mL of media and the number of live cells was determined using a Nexcelom Auto 2000 Cellometer (immune cells program, low RBC) following ViaStain AOPI (acridine orange and propidium iodide) viability staining (Nexcelom cat. #CS2-D106-5ML). Cells were plated at 10,000–15,000 live cells per cm^2^ on tissue culture treated plastic (CytoOne 6-well plates, #CC7682-7506).

### 2.4. Optimization of MSC Expansion

Our previously described method MSC expansion medium was the standard used for comparison. Since that medium contains more than 10 components [24], our goal was to reduce the number of medium components while maintaining the MSC attachment at isolation/startup and maintaining MSC expansion, CFU-F efficiency, trilineage differentiation potential, MSC surface marker expression, and cellular morphology similar to or better than that standard. Here, low glucose Dulbecco's Modified Eagle's Medium (DMEM Life Technologies cat. #14190) supplemented with 1% GlutaMAX (Life Technologies cat. #35050), with 1% Antibiotic-Antimycotic, and, by volume, with 2, 5, or 10% pooled human platelet lysate (HPL, pooled from more than 25 outdated platelet donors, supplied by Kansas University Medical Center diagnostic laboratory, Dr. Lowell Tilzer, director) and 4 units/mL heparin was tested. The cells were plated at 10–15,000 cells per cm^2^ in CytoOne flat bottom tissue culture treated 6-well plates and expanded for 5 passages. Cells were incubated and grown as a monolayer at 37°C, 5% CO_2_, and 90% humidity (Nuaire AutoFlow 4950 or Heracell 150i). Once the cells reached approximately 80–90% confluence they were lifted and plated in fresh medium. To lift the cells, the medium was removed and cells were washed with 37°C DPBS. The DPBS was removed and replaced with 37°C 0.05% trypsin-EDTA (Lifetech #25200-056). Following a 3–5-minute incubation at 37°C, the plates were tapped to release cells and the enzymatic digestion was terminated with 3 volumes of media. Cells were pelleted at 200 ×g for 5 minutes at room temperature. Supernatant was discarded and 1 mL of media was used to suspend the cells. Cells were counted using the Nexcelom Auto 2000 Cellometer and the ViaStain AOPI staining reagent using the manufacturer's protocol and a built-in settings. At passage, the number of cells, percentage of live cells, cell size, and number of hours in culture were recorded. Cells were initially plated at a density of approximately 10,000 cells/cm^2^; using this as the initial cell number and the number of cells at harvest as the final cell number and culture time, population doubling time was calculated using the standard formula. At times, extra cells were frozen for later use. To freeze, cells were cryopreserved using a 1 : 1 ratio of HPL media and cryopreservative (Globalstem #GSM-4200) and held on ice until transfer to a controlled rate freezing device (Mr. Frosty) and being placed into a −80°C freezer overnight. The next day, the vials were moved to the vapor phase of liquid nitrogen for long term storage.

### 2.5. CFU-F Assay

MSCs were plated at 10, 50, or 100 cells per cm^2^ in duplicate in 6-well CytoOne tissue culture plates in 2, 5, and 10% HPL enriched DMEM, as described above. Four cell lines were expanded 4 days in culture, prior to fixation and methylene blue staining. Subsequent tests used 4–7 days of culture at a density of 5, 10, or 50 cells per cm^2^. After the required culture period, the medium was removed and the cells were washed with DPBS and then fixed using 4°C 100% methanol for 5 minutes. The cells were washed again with DPBS, stained with 0.5% methylene blue for 15 minutes, rinsed several times with distilled water, and air dried. The stained colonies were counted manually at 40x final magnification. Colonies were defined as isolated groups (clonal groups) of at least 10 cells. Colony number was determined by averaging the number of colonies in the technical replicates at each plating density for a given expansion period. Colony forming efficiency was calculated by dividing the number of plated cells by the number of colonies.

### 2.6. Differentiation

Differentiation of MSCs was induced by replacing the expansion medium with MSC differentiation medium (StemPro, Life Technologies #s A10070-01, A10071-01, and A10072-01 for adipogenic, chondrogenic, and osteogenic differentiation) and following the manufacturer's protocol. After about 21 days of differentiation, the differentiation medium was removed; the MSCs were washed with DPBS, fixed with 4% paraformaldehyde for 10 min, and stained with Oil Red for analysis of adipose cells, Safranin O for chondrogenic cells, or Alizarin Red S for osteogenic cells. Micrographs were taken using an Evos FL Auto microscope (Life Technologies).

### 2.7. Flow Cytometry

The BD human MSCs flow cytometry characterization kit was used for positive and negative surface marker staining (#562245). Using the manufacturer's recommended protocol, MSC samples were stained with four fluorochromes together including positive and negative staining cocktails. The positive marker cocktail stained for CD90, CD105, and CD73 (defined as >97% positive staining). The negative cocktail (all antibodies were stained using a single fluorochrome, PE) stained for CD34, CD45, CD11b, CD19, and HLA-DR (defined as <2% positive staining). A CD44 labeled PE antibody was used as positive control for the negative cocktail to set the compensation and gating of the negative cocktail. For each flow cytometry run, fluorescence minus one controls for each fluorochrome and isotype controls for each antibody were used for compensation and nonspecific fluorescence analysis. Samples were washed with 1% BSA solution before and after staining. A FACScalibur (BD Biosciences) was used for flow cytometry and analysis was conducted using FCS software. Negative staining gate of the isotype control was set at 1% positive staining.

### 2.8. Statistics

After confirmation that ANOVA assumptions of normality and homogeneity of variance were met, ANOVA was used to evaluate significant differences between optimization variables. If the assumptions were violated, the dataset was transformed mathematically and again tested to see if it met ANOVA assumptions. Hypothesis testing was two tailed (e.g., mean 1 ≠ mean 2). After running ANOVA and finding significant main effect(s) or interaction terms,* post hoc* means testing of planned comparisons was conducted using either the Bonferroni correction or Holm-Sidak method. Significance was set at *p* < 0.05. Data is presented as average (mean) plus/minus one standard error. In one case, in order to pass the normality test (Shapiro-Wilk) an “outlier” was removed. After the outlier was removed, the dataset passed the normality test and ANOVA determined that there was a significant effect of HPL concentration. SigmaPlot v.12.5 (Systat software) was used for statistics and making of the graphs. The graphs created in SigmaPlot were saved as EPS files and moved into a vector-based graphics package (Adobe InDesign or Adobe Illustrator CS6) for editing and rendering.

## 3. Results

### 3.1. Umbilical Cords

Umbilical cord from Caesarean-section delivery (*n* = 17) and “normal” vaginal delivery (*n* = 7) were used in this research. The biographic data of each cord is shown in Table 1.

### 3.2. Isolation Method Comparison

**Figure 2:**
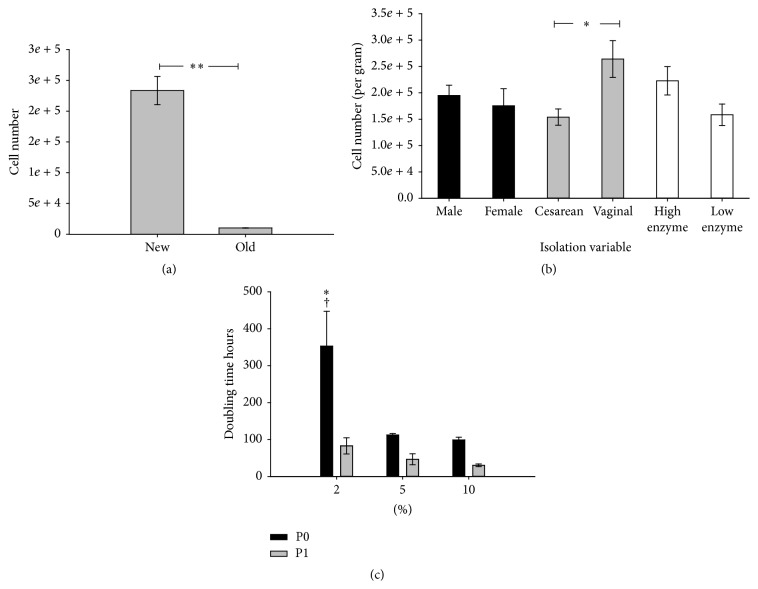
Effect of various experimental variables on UC-MSCs isolation. (a) The new methods average cell number per cm of umbilical cord isolated, compared to the old cell number isolated per cm (*∗∗* means *p* < 0.001). (b) Comparing different experimental variables. Significant difference observed in Caesarean-section delivery versus vaginal delivery (*∗* means *p* < 0.05). (c) Population doubling time for passage 0 (initial isolation) or passage 1 (first passage of expansion phase). *∗* represents *p* < 0.05 for 2% hpl media compared to 5% and 10% media. † represents *p* < 0.05 for the passages (P0 compared to the P1).

**Figure 3:**
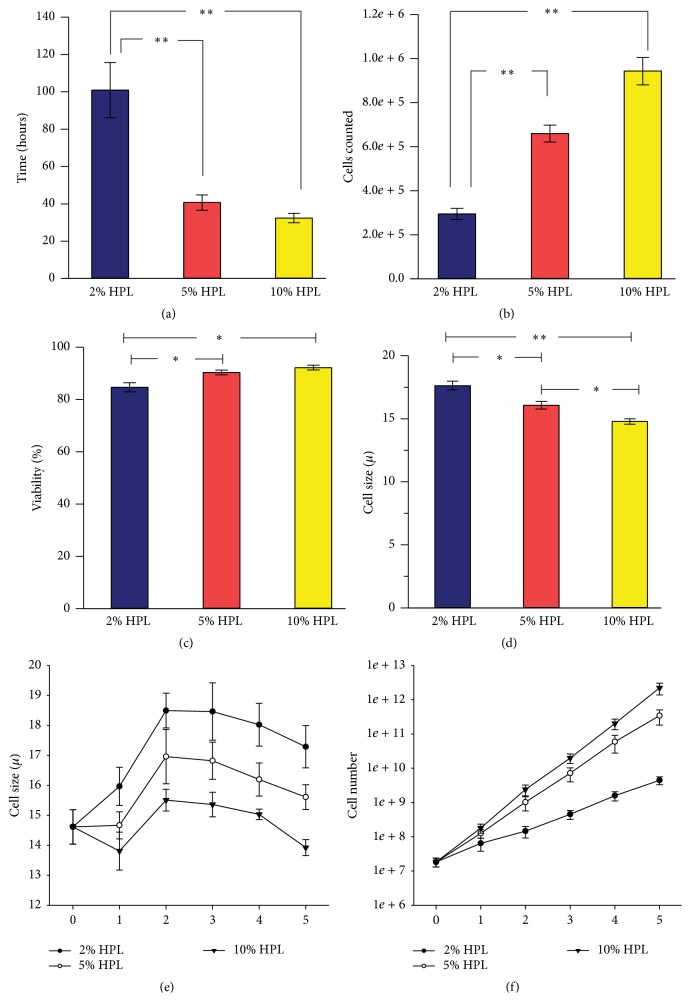
Effect of HPL concentration on expansion. (a–d) UC-MSC (*n* = 6) expansion results combined for passages 1–5. (a) Population doubling times for the 3 media conditions. (b) Number of cells counted at passage for each media condition. (c) Cell viability at passage for each media condition. (d) The average size of the cell for each media condition at passage. (e) Cell size over 5 passages for each media condition. (f) The theoretical yield if an entire umbilical cord was isolated and grown to confluence at each passage. *∗* means *p* < 0.05 and *∗∗* means *p* < 0.001.

Note that the MSC expansion comparison was considered for passages 1–5, and passage 0 was considered part of the isolation of MSCs. Results obtained from our previously described method (historical data from 27 umbilical cords [24]) and our optimized method were compared. As shown in Figure 2, the optimized method yielded on average 10 times more MSCs per cm length than the original method and yielded MSCs in 100% of the UC samples. Note that in pilot work where we were identifying variables to optimize, we did fail to isolate MSCs in two cases. But even in these cases, MSCs were isolated from the same UC in different samples (e.g., in no cases did we suspect that the UC did not contain viable MSCs). While we did not test for bacterial, viral, or fungal contamination, no break in sterility was apparent here (e.g., no frank contamination was observed and no cultures were discarded due to contamination). Live cells per cm of length or per gram were compared in Table 1; there was a trend for the coefficient of variation to be less for live cells per gram. The optimized method uses a closed processing system for tissue disruption and takes a total of 4 hours of work time plus a 3-hour enzyme extraction step to isolate the umbilical cord MSCs. MSC attachment was observed within 24 hours of the isolation and proliferation was observed in all three HPL media enrichment conditions. As shown in Figure 2(c), during the isolation phase (P0) UC-MSCs grew more quickly when plated in 5% or 10% HPL enriched DMEM than UC-MSCs plated in 2% HPL enriched DMEM. It is possible that UC-MSCs grown in 5 or 10% HPL enriched DMEM attached more quickly than those grown in 2% HPL enriched DMEM in P0. The growth rate difference for 2% HPL enriched medium was statistically different (slower) at P0 then later passages (see Figures 2(c) and 3(a)) and was significantly different (slower) than 5 and 10% HPL enriched media at isolation and during expansion.

### 3.3. MSC Expansion Comparison

Note that the MSC expansion comparison was considered for passages 1–5, and passage 0 was considered part of the isolation of MSCs. UC-MSCs were expanded for passages 1–5 here. UC-MSCs were evaluated in 3 different growth conditions: DMEM supplemented with 2% HPL, 5% HPL, or 10% HPL. A two-way ANOVA (main effects HPL level and expansion over time) found a significant main effect (HPL concentration) on attachment and expansion. In* post hoc* testing, we found significantly more cells—about 30% more were obtained when cells were expanded in 10% HPL enriched DMEM medium compared to 5% HPL enriched medium (9.4 × 10^5^ ± 6.2 × 10^4^ cells per cm^2^ versus 6.6 × 10^5^ ± 3.8 × 10^4^ for 5% HPL enriched medium (Figure 3(b)). Similarly,* post hoc* testing showed significantly shorter population doubling times when MSCs were expanded in 10% HPL (32.4 ± 2.5 hours), compared to 40.7 ± 4.1 hours for 5% HPL and 100.9 ± 14.8 hours for 2% HPL enriched medium (shown in Figure 3(c)). As shown in Figure 3(d), MSCs grown in 10% HPL enriched DMEM averaged 17% smaller than those grown in 2% HPL (14.7 ± 0.2 *μ*m versus 17.6 ± 0.4 *μ*m) and 10% smaller than cell grown in 5% HPL enriched medium (on average over 5 passages, 16.1 ± 0.3 *μ*m). The trends in MSC size across HPL medium conditions became noticeable after the second passage (Figure 3(e)). HPL medium enrichment affected the viability of the cells noted at passage (see Figure 3(c)). Subtle but significant differences were found in viability at passage between the three medium conditions: MSCs in expanded in DMEM supplemented with 10% HPL had higher viability than those grown in DMEM supplemented with 2% HPL (92.2 ± 0.9% versus 84.9 ± 1.7%) and 5% HPL supplemented medium had significantly greater viability than 2% HPL medium (90.4 ± 0.9%; see Figure 3(c)).

The theoretical cell yield was calculated assuming the entire umbilical cord was isolated and expanded in each medium condition to passage 5. As shown in Figure 3(f), it was estimated that the total yield might exceed 10^12^ MSCs (a trillion cells) at passage 5 for UC-MSCs expanded in 10% HPL supplemented medium and exceed 10^11^ MSCs for UC-MSCs expanded in medium supplemented with 5% HPL (Figure 3(f)).

### 3.4. Evaluation of UC-MSC Characteristics

Sex of the donor had no effect on number of MSCs isolated (Figure 2(b)), or the estimated number of MSCs obtained after expansion (data not shown). In contrast, a significant increase in the number of cells isolated was found for UC-MSCs isolated from normal vaginal delivery compared to those collected following Caesarean-section delivery (see Figure 2(b)).

### 3.5. Colony Forming Unit-Fibroblast (CFU-F) Data is Presented as a Normalized Unit: Colony Forming Efficiency (CFE; CFE = Number of Plated Cells Divided by Number of Colonies)

**Figure 4:**
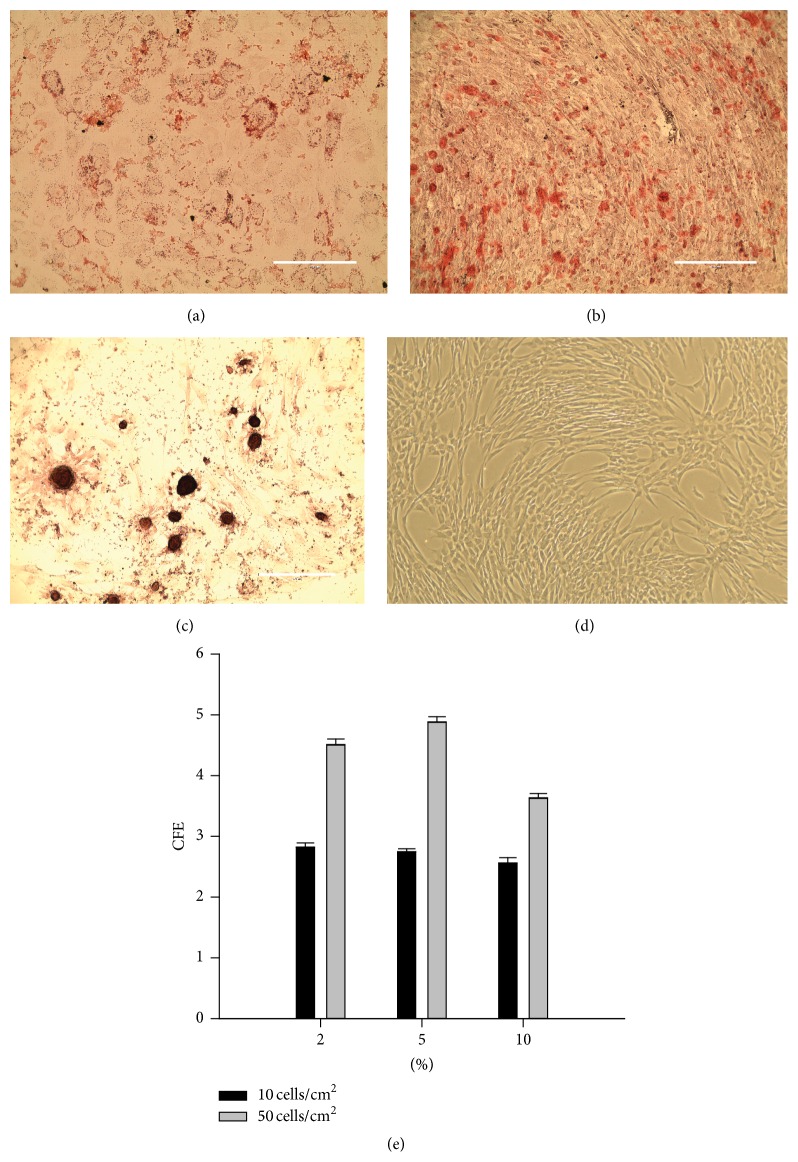
Differentiation and colony forming unit fibroblast (CFU-F) results for the characterization of UC-MSCs. (a) After adipogenic differentiation, MSCs were stained with Oil Red which binds to lipid droplets (20x objective magnification; scale bar = 200 micrometers). (b) After osteogenic differentiation, MSCs were stained with Alizarin Red S which binds to calcium deposits. (c) After chondrogenic differentiation, MSCs stained with Safranin O which binds to glycosaminoglycans in cartilage ((b) and (c) at 10x objective magnification; scale bar = 400 micrometers). (d) UC-MSCs in normal growth conditions (control) phase contrast micrograph at 4x objective magnification. (e) CFU-F efficiency was calculated by dividing the number of plated cells by the number of CFU-F colonies observed. Panel (e) shows colony forming efficiency versus human pooled platelet lysate (HPL) concentration in medium (2, 5, or 10% HPL) after plating at 5 (black bars) or 10 (gray bars) cells per cm^2^ and 4 days in culture.

As shown in Figure 4(e), the concentration of HPL supplementation had no effect on CFE at 10 cells/cm^2^ (100 cells per well of a 6-well plate) after 4 days of culture. In contrast, when plated at a density of 50 cells per cm^2^ and 4 days of expansion in culture, 10% HPL supplementation resulted in an increased colony forming efficiency compared to 2 and 5% HPL: 2–4 MSCs were needed to form a colony when plated in medium supplemented with 10% HPL (Figure 4(e)). As seen in Figure 4(e) and as previously reported [26, 27], plating density affects colony forming efficiency and higher efficiency is found at lower plating density. Therefore we determine whether higher efficiency would be found at plating density below 10 cells/cm^2^ after plating in HPL. The highest colony forming efficiency was found when MSCs were plated at 5 cells/cm^2^ for 6 days (50 cells per well of a six-well plate); in medium supplemented with 10% HPL: on average one out of two MSCs formed a colony (see Supplemental Figure 1 in Supplementary Material available online at http://dx.doi.org/10.1155/2016/6810980).

### 3.6. Differentiation

MSCs isolated and expanded using the optimized method undergo differentiation to the three mesenchymal lineages, bone, cartilage, and fat after exposure to differentiation medium conditions for 3 weeks.  Figure 4 shows MSCs differentiated to fat and chondrogenic and osteogenic lineages following closed isolation method and expansion to passage 5 in 10% HPL supplemented DMEM. Exposure to adipogenic differentiation medium resulted in formation of lipid droplets in MSCs that stained with Oil Red (Figure 4(a)). Exposure to osteogenic differentiation conditions resulted in calcium deposits formed within MSCs which stained with Alizarin Red S (Figure 4(b)). Cartilage-like tissue formation was observed in clusters of cells after exposure to differentiation medium as indicated by glycosaminoglycan staining by Safranin O for chondrogenic cells (Figure 4(c)).

### 3.7. Flow Cytometry

**Figure 5:**
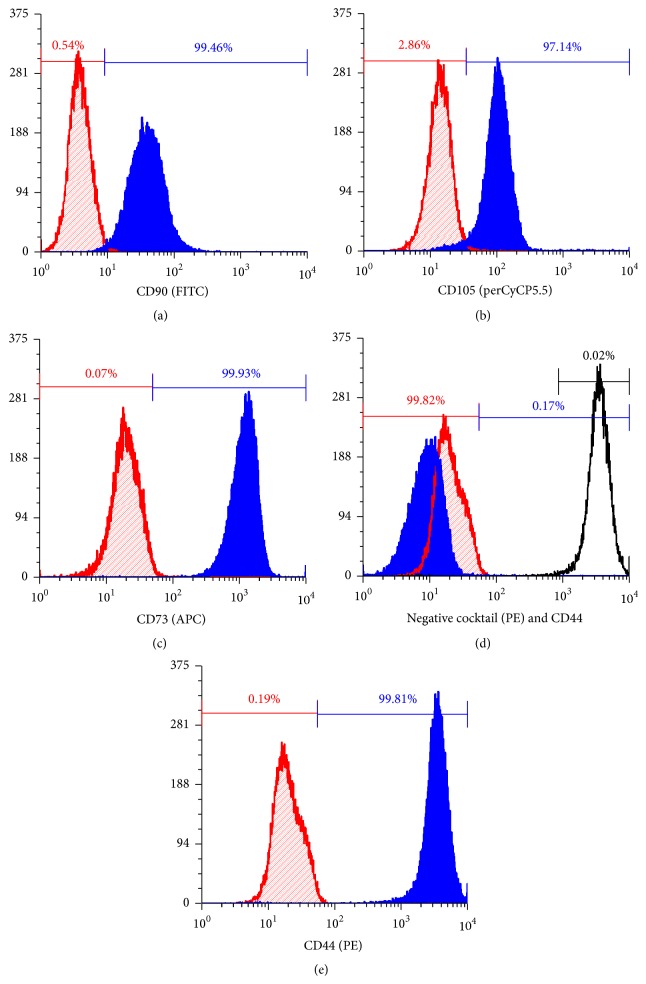
Histograms of flow cytometry; blue solid filled overlay represents the test sample; red diagonal line filled overlay represents the isotype control. For each histogram the negative gate (red bar) was set for inclusion of 99% of the isotype. Percentages shown in histograms are for the test samples. (a–c) All are markers in the positive cocktail, CD90, CD105, and CD73. The positive gate percentages shown in blue for each sample. (d) The negative cocktail, with CD44 included as a positive control (unfilled overlay) positive percentage in black. (e) CD44 marker included outside of the positive cocktail.

Flow cytometry was used to analyze the surface marker expression in 5 MSC lines following isolation using the closed processing protocol and expansion using the 10% HPL supplemented DMEM for 5 passages. High expression (>95% positive) for surface markers CD73, CD90, CD105, and CD44 was observed (Figure 5 for representative results, Supplemental Table 1 for all flow cytometry data). Low surface maker expression (<0.5% positive) was observed for CD34, CD45, CD11b, CD19, and HLA-DRT (Supplemental Table 1). To evaluate the effect of freezing and thawing MSCs on surface marker expression, four MSCs lines were evaluated before and after a freeze/thaw cycle. No significant differences were found in surface marker expression between frozen/thawed and never frozen MSCs in surface marker expression (Supplemental Table 2).

## 4. Discussion

The acceleration of stem cell and regenerative medicine clinical trials, and MSC trials in particular, has produced a renewed effort to standardize production and characterization of MSCs in GMP-compliant SOPs. Umbilical cord MSCs have a number of advantages which suggest that they might be an important source for allogeneic MSCs for cellular therapy, and, as indicated by trends in MSC clinical trials worldwide, this MSC source is a needed one.

In order to develop an SOP for GMP production of UC-MSCs, we identified limitations in our previously described method for UC-MSC isolation and expansion that represented barriers for GMP production. First, our previous isolation method required a lengthy dissection step and the opening of the umbilical cord and manually removing the vessels prior to mincing the Wharton's jelly was time consuming and increased contamination risk. Here, we sought to reduce processing time and reduce contamination risks. We reasoned that a standardized method for liberating MSCs from the Wharton's jelly may produce a more homogenous product. Second, the previously described UC-MSC medium, which was originally described by Catherine Verfaillie's lab for expansion of MAPCs, is complicated with more than 10 components and it contained 2% FBS, a xenogeneic product [28]. We sought to identify a simplified medium that could be free of xenogeneic materials and contain fewer components. We tested human platelet lysate (HPL) enriched medium. Previous work indicated that HPL could be produced in a GMP-compliant format and has been reported to produce good expansion of MSCs [29–31]. Here, we found that 5 or 10% HPL enrichment vastly improved MSC expansion in the P0 (initial isolation). Furthermore, we found robust expansion over passages 1–5. Therefore, use of HPL-enriched medium eliminated two barriers to GMP-compliant manufacturing of UC-MSCs. However, using a pooled human blood product is not without certain risks, they have been somewhat mitigated (discussed below). Due to the sample to sample variability, pooling of platelet lysate is essential to produce a uniform product [32]. For example, human pathogens that escape screening by the providers may contaminate HPL samples. One possible way to address this risk would be to inactivate pathogens in HPL [33]. We did not inactivate pathogens in our pooled HPL, but the repeated freeze-thaw process followed by the filtration through a 0.2 um filter should remove all potential bacteria and parasites. While gamma irradiation is something that may be considered to lower viral risk, the blood products used were obtained from a blood bank for clinical use and thus had met all existing blood screening safety measures.

Here, 1 cm sections of cord were used to optimize the protocol. The one cm length sections of umbilical cord provided enough cells for isolation and expansion, and many technical replicates were available from one cord which allows multiple experimental variables to be examined in each cord (the biological variable). We assumed that a randomly selected, one cm length of cord would adequately represent the umbilical cord (e.g., that cellular distribution and umbilical cord extracellular matrix are homogenous). This assumption was not validated by us, nor do we know of any investigation that supports or denies this assumption. Umbilical cords display tremendous biological variation in density (weight per unit length), diameter, and physical mechanical properties perhaps due to the amount of extracellular matrix surrounding the vessels (see Table 1). The gram per cm measurements vary within each technical replicate from a single cord and between different umbilical cords, too. This indicates the importance for multiple biological and technical replicates when performing optimization testing using umbilical cords. The number of cells varied considerably between each umbilical cord as did the density and amount of extracellular matrix. Thus the biological variation limits the ability to manufacture a standard cellular therapy product. For example, does the physiology of MSCs vary between umbilical cords and how can we optimize the clinical effect of MSCs? We have assumed that the cells isolated after an initial passage are similar in physiology, but this assumption will require more assaying to confirm.

Several protocols for isolation of MSCs from different parts of the umbilical cord have been published [26, 34, 35]. These protocols require dissection of different portions of the umbilical cord and a variety of methods to enrich MSCs from the primary isolation population. This contributes to variation in the number of cells in the primary isolation and their ability to undergo expansion in culture. We did not observe frank differences in the population of MSCs isolated following their extraction from Wharton's jelly versus those isolates following disruption of the entire 1 cm cord fragment. We found that extraction of the entire 1 cm length using the methods outlined here gave a >10-fold increase in the number of input cells for the primary culture. We attribute the reduced manipulation of the tissues (elaborate dissection negatively affects the attachment and expansion) and the more efficient removal of red blood cell contamination (blood negatively affects the viability, attachment, and expansion of MSCs) to the improved extraction and expansion efficiency.

Previously we used “cord length” measurement for comparisons of yield between cords. Here, we tracked both length and weight to determine whether either proved to be a better predictor of cell yield in initial isolation. The variation between umbilical cords for both length and weight is represented in Table 1. As seen in Table 1, weight was a more reliable measurement compared to cord length. Additional work is needed to determine differences between the predicted total cell yield from an umbilical cord and the estimated value. We have not processed cells from an entire umbilical cord and therefore cannot confirm the accuracy of these estimates. Our current method is readily scaled up, and so this information is forthcoming.

The increase in cell numbers from the optimized protocol may be attributed to the faster processing and reduced dissection when isolating the MSCs. By not removing the blood vessels, the optimized method is a significant departure from our previously described method. Therefore, this casts into doubt whether the same cell population has been isolated, and whether the MSCs obtained using the optimized methods are similar or different from those obtained using the previous methods. As mentioned in Section 1, several different methods for obtaining MSCs from the umbilical cord have been described, and it is unclear whether each isolates the same cells. Our data does not directly address this question, but we demonstrate here that following evaluation of 5 umbilical cord MSC isolates; the cells isolated from the optimized methods conform to ISCT criteria for MSCs [4].

As far as we know this paper is the first to demonstrate a difference between MSC isolation efficiency from umbilical cords derived from vaginal births versus Caesarean-section births. Vaginal birth umbilical cords had more cells per after isolation by approximately 41% (Figure 2(b)). Prior to observation, we preferred to use Caesarean-section umbilical cords for MSC isolation because we assumed that surgical collection would have a reduced contamination risk compared to cord collected following the passage through the birth canal. During this study we found no differences in contamination from either vaginal birth or Caesarean-section umbilical cords. Similar to our observations about MSCs from vaginal versus Caesarean-section cords, the volume of umbilical cord blood collected is decreased by vaginal birth over Caesarean-section birth [36, 37]. We did not see a sex difference between the number of cells isolated or MSC expansion rate or number. Enzymatic digestion using a high concentration of digestive enzymes tended to have higher yield at isolation (Figure 2(b)). Visually, higher concentration of enzyme samples appeared to have less debris when compared at initial plating compared to lower enzyme concentration.

After the initial plating of cells during the isolation protocol, there is a delay in cell attachment. Attachment to the substrate is a defining characteristic of MSCs and appears to be necessary for MSCs expansion. We noted that after the isolation of MSCs, in passages 1–5, MSCs attach and begin to expand within 24 hours of plating. In contrast, the time to reach the confluence for the isolation and initial passage is significantly impacted by delays in attachment. Here, we included the P0 data with the isolation of MSCs and considered passages 1 through 5 for the expansion phase of MSC characterization. We noticed a trend that when there was a higher viability at the initial isolation, the cells attached better and expanded more rapidly. In our prior work, cell viability was not recorded at the initial isolation. Here, the use of the Nexcelom and ViaStain AOPI viability assay provided a quantitative method and gave more consistent results than trypan blue and manual counting using the haemocytometer (which is how we counted cells, previously). Automated cell counting lends itself to optimization and producing SOPs.

When considering the production of a public bank of cord samples, freezing the primary isolates at P0 → P1 is likely to be a necessity. Others have reported this affects cells surface marker expression or viability [38]. For that reason, we evaluated surface marker expression in never frozen cells and in cells subjected to a freeze/thaw cycle. The flow cytometry analysis did not show a difference in surface marker expression between fresh cells (e.g., those never frozen) and cells frozen and thawed cells for four umbilical cord MSCs lines. Future testing is needed to confirm that these results stand up with a larger sample size. Clinical trials will require the freezing of cells for use, the use of fresh cells in clinical trials is not feasible when considering the rigorous quality control and release testing that must be done to determine if these cells meet the standards for clinical use.

Here, human platelet lysate enriched media at three different concentrations (2%, 5%, and 10%) was used to analyze the effect on the initial isolation (P0) and growth of the MSCs for passages 1–5. We evaluated MSCs through passage 5 to characterize expansion potential. We observed that P0 to P1 expansion exhibited the highest amount of variation in growth rate (see Figures 2(c) and 3(a)). Results from six umbilical cords did not show a difference between passages 1–5 for population doubling time, number of cells at passage, and viability at passage (data not shown). However, the three media conditions did affect these variables. Enrichment with 2% HPL enriched medium was significantly different than 5% and 10% HPL enriched media for population doubling, cell size, cell numbers, and viability. Enrichment with 2% HPL had slower population doubling, fewer cells at passage, larger cells, and a lower percentage of viable cells at passage. We observed a trend associated with better results for these measurements as HPL enrichment in the medium increased. For this reason we chose 10% as the new standard media condition to be used to grow the UC-MSCs. Cell size for the UC-MSCs was a variable we did not expect to vary significantly between the different media conditions, but we observed a significant difference in cell size with higher HPL concentration. We noticed a trend for cell size to initially increase after the first passage and then decrease over subsequent passages in all media conditions (Figures 3(d)-3(e)). We cannot explain this observation. Further work is needed to assess whether cell size is affected by passage, since we have previously observed that senescent cells are larger, and because we would expect an increase in cellular senescence with passage. If the contrary is true, for example, the fact that more rapidly dividing cells are smaller, then the cell size data could support our conclusion that 10% HPL is the optimal growth condition for the UC-MSCs. Previous work had indicated that smaller MSCs with a rapidly dividing phenotype could be identified by plating at a density of 3 cells per cm^2^ [39]. Here, we did not evaluate the effect of plating density on proliferation. Future work should evaluate the interaction between plating density and 10% HPL media to optimize manufacturing efficiency.

MSC characterization was done by assessing cell surface markers with flow cytometry, CFU-F, and differentiation capacity. All five cell lines we analyzed with flow cytometry had high levels of the surface markers known to be associated with MSCs. The high percentage of positive cells (>95%) is comparable to the previously published method [24]. These results suggest a homogenous cell population was isolated even though the blood vessels were not removed for the isolation step. Differentiation ability was assessed in the same five cell lines and all display trilineage differentiation capacity. The capacity for adipogenic differentiation was analyzed by Oil Red O staining for lipid droplet accumulation within the differentiated cells cytoplasm. Analysis showed multiple lipid droplets forming within a large number of the cells (Figure 4(a)). Cell death did occur during the time to differentiate, leading to space between the adipogenic cells in the figure. Osteogenic differentiation was stained with Alizarin Red S to analyze calcium deposit formation. Staining was observed in calcium deposits on the cells and within the cells as seen in Figure 4(b). Chondrogenic differentiated cells were stained with Safranin O to assess if cartilaginous associated with glycosaminoglycan. This differentiation yielded circular colonies, often remaining adhered to the plate and they robustly stained for Safranin O. Typically histological sections of microcolonies are used to assess chondrogenic lineage differentiation. Our results indicate this is not necessary when small colonies of cell remain adherent (Figure 4(c)). Although the results are not quantified, the quality of the staining and duplication between multiple lines provides good evidence that MSCs isolated and expanded by the new method have robust trilineage differentiation potential.

Colony forming unit fibroblast (CFU-F) efficiency analyzed self-renewal potential of UC-MSCs. Compared to previous research for UC-MSCs expanded in 21% oxygen, fewer cells were needed to form a colony using the method described here, suggesting a higher colony forming efficiency [27]. We considered the 10 cells per cm^2^ a more reliable measure for the effect of HPL concentration due to difficulty counting cells at 50 cells per cm^2^. The fast growth rate for the 10% HPL enriched medium made our previous CFU-F protocols unreliable because the plates grew too fast. We tested growth conditions for measuring colony forming efficiency by analyzing days from plating versus colony counts. We found the number of cells to form colonies decreased with each day of growth; the exception was 50 cells per cm^2^ which increased. The highest CFU-F efficiency was for 5 cells per cm^2^ cells grown for 6 days. We determined using both 10 and 50 cells per cm^2^ yield consistent data for CFU-F. The self-renewal data (colony forming efficiency) is important when estimating the expansion potential of a MSC line. Higher CFU-F efficiencies are associated with MSC lines displaying a more robust growth potential. Determining the method for analyzing CFU-Fs in these fast growing cells allows for analysis of growth potential for future research using UC-MSCs.

Here we provide a new, optimized method to isolate and expand UC-MSCs. When compared to our previous method, an increase in total MSC yield at the initial isolation of more than 10 times was obtained, and less time is needed to isolate MSCs from the umbilical cord. Additionally, this method reduces the overall expansion by reducing the amount of population doubling needed to meet our production target of 2–10 billion cells per batch. The method uses closed system for initial isolation with minimal dissection of the cord and, thus, reduces contamination risk, while simultaneously reducing processing time. The method uses a simplified (5 component), xenogen-free medium that can be upgraded to GMP-compliant components for scale-up. Characterization of MSCs produced using this optimized processing protocol and simplified medium included* in vitro* expansion, colony forming efficiency, and trilineage differentiation to osteogenic, chondrogenic, and adipogenic lineages, and surface marker expression by flow cytometry indicates that MSCs were produced by this method. Further work is needed to confirm that MSCs isolated and expanded using this method will perform* in vivo* as a cellular therapy. We are developing an* in vivo* model to test the potency of MSCs which may serve as suitable assay to compare the potency of various manipulations such as “priming” or licensing of MSCs. Taken together, this work will speed clinical translation of UC-MSCs by providing the basis of chemistry and manufacturing controls (CMC) portion of an investigation of new drug application (IND).

## 5. Conclusion

The methods we developed for isolation and expansion of UC-MSCs address some challenges to translation to clinical use. We report an increased MSC yield for vaginal births compared to Caesarean-section births. The new isolation method provides the necessary cell yield for banking and uses a closed system that can be easily scaled up and expansion media supplemented with 10% HPL had the best growth rate. These results provide improvements which may support GMP manufacturing of UC-MSCs. To complete the validation of this new method, functional testing for immune modulation or regenerative potential testing is needed.

## Supplementary Material

The supplemental figures show the surface marker expression for six UC-MSC isolates and additional CFU-F information. Table 1 contains the surface marker expression data for six isolates. Note that all isolates conform to ISCT MSC surface marker criteria. In Table 2, the surface marker expression of UC-MSC isolates which have been frozen and never frozen was compared. Note that freezing did not significantly impact surface marker expression. Supplemental Figure 1 shows CFU-F expression of UC-MSCs evaluated at different times after plating. Based upon this experiment, day 6 at 5 cell per cm^2^ appears to yield the highest colony forming efficiency.

## Figures and Tables

**Table 1:** Data from 24 umbilical cord MSC isolations.

UC-MSC line	Sex	Birth	Length (cm)	Enzyme	Weight (g)	Gram per cm	Viability	±SE	Live cells per cm	±SE	Live cells per gram	±SE	Theoretical cell yield
241	F	V	46	High	67.9	1.5	76.7%	0.6%	3.7*E* + 05	2.3*E* + 05	3.2*E* + 05	5.6*E* + 04	2.17*E* + 07
242	M	V	43	High	60.7	1.4	58.0%	1.9%	2.4*E* + 05	2.9*E* + 04	1.7*E* + 05	2.2*E* + 04	1.06*E* + 07
243	F	V	57	High	81.1	1.4	62.8%	2.0%	3.9*E* + 05	7.4*E* + 04	2.7*E* + 05	6.4*E* + 04	2.17*E* + 07
244	M	C-S	35	High	66.0	1.9	58.6%	2.6%	2.0*E* + 05	2.4*E* + 04	1.2*E* + 05	1.8*E* + 04	7.85*E* + 06
245	M	V	61	High	76.0	1.2	50.6%	6.4%	3.3*E* + 05	9.9*E* + 04	2.7*E* + 05	9.2*E* + 04	2.06*E* + 07
246	M	C-S	41	High	60.9	1.5	66.0%	0.9%	1.5*E* + 05	4.3*E* + 03	8.5*E* + 04	8.2*E* + 03	5.19*E* + 06
248	F	C-S	47	High	72.6	1.5	68.3%	3.2%	2.9*E* + 05	4.2*E* + 04	1.2*E* + 05	4.3*E* + 04	8.66*E* + 06
249	F	C-S	32	High	37.7	1.2	64.2%	10.7%	1.3*E* + 05	4.9*E* + 04	1.2*E* + 05	4.3*E* + 04	4.50*E* + 06
250	M	V	26	High	43.3	1.7	84.2%	0.5%	5.3*E* + 05	4.1*E* + 04	3.1*E* + 05	8.8*E* + 03	1.34*E* + 07
251	F	V	28	High	30.4	1.1	74.4%	3.8%	9.9*E* + 04	5.7*E* + 02	8.8*E* + 04	1.7*E* + 04	2.69*E* + 06
252	M	C-S	54	High	83.6	1.5	79.8%	3.9%	1.9*E* + 05	3.9*E* + 04	1.3*E* + 05	5.0*E* + 04	1.11*E* + 07
253	M	V	61	Low	48.8	0.8	58.0%	3.7%	3.4*E* + 05	9.8*E* + 04	2.3*E* + 05	6.5*E* + 04	1.11*E* + 07
254	F	C-S	38	Low	59.3	1.6	60.9%	3.9%	1.1*E* + 05	5.3*E* + 04	7.9*E* + 04	4.0*E* + 04	4.68*E* + 06
255	F	C-S	47	Low	82.3	1.8	75.1%	8.6%	7.7*E* + 04	5.2*E* + 03	4.7*E* + 04	4.0*E* + 03	3.85*E* + 06
256	M	C-S	45	Low	49.0	1.1	70.2%	1.1%	2.4*E* + 05	5.8*E* + 04	2.1*E* + 05	3.3*E* + 04	1.02*E* + 07
257	M	C-S	43	Low	105.2	2.4	66.6%	3.0%	3.5*E* + 05	2.4*E* + 04	1.5*E* + 05	1.4*E* + 04	1.56*E* + 07
258	M	C-S	37	Low	45.1	1.2	62.5%	4.6%	1.9*E* + 05	3.8*E* + 04	1.6*E* + 05	2.7*E* + 04	7.01*E* + 06
259	M	C-S	31	Low	57.8	1.9	62.2%	3.5%	2.7*E* + 05	2.6*E* + 04	1.5*E* + 05	2.2*E* + 04	8.56*E* + 06
260	F	C-S	67	Low	100.6	1.5	64.4%	1.8%	1.7*E* + 05	3.9*E* + 04	1.1*E* + 05	2.8*E* + 04	1.11*E* + 07
261	M	C-S	51	Low	92.8	1.8	72.3%	2.1%	2.2*E* + 05	1.9*E* + 04	1.2*E* + 05	8.8*E* + 03	1.10*E* + 07
262	F	C-S	28.5	Low	25.1	0.9	50.5%	4.1%	1.0*E* + 05	2.7*E* + 04	1.5*E* + 05	5.7*E* + 04	3.81*E* + 06
263	F	C-S	28	Low	23.8	0.9	65.3%	3.8%	2.0*E* + 05	5.4*E* + 04	2.6*E* + 05	8.9*E* + 04	6.27*E* + 06
264	F	C-S	32	Low	45.7	1.4	59.8%	3.4%	4.5*E* + 05	4.7*E* + 04	3.2*E* + 05	4.3*E* + 04	1.46*E* + 07
265	M	C-S	38	Low	38.7	1.0	54.8%	3.2%	1.5*E* + 05	3.0*E* + 04	1.5*E* + 05	3.5*E* + 04	5.81*E* + 06

All 24 cords were compared below													

			Length		Weight	g per cm	Viability		Live cells per cm		Live cells per gram		Theo. yield

Mean			42.4		60.6	1.4	65.25%		2.42*E* + 05		1.72*E* + 05		1.01*E* + 07
Standard dev.			11.6		22.9	0.4	8.33%		1.16*E* + 05		7.48*E* + 04		5.00*E* + 06
Coefficiency of var.			27.5%		37.7%	26.9%	12.8%		47.9%		43.5%		49.7%

F = female, M = male, C-S = Caesarean-section, and V = vaginal. The enzyme concentration: low was 300 U/mL and high was 532 U/mL of collagenase. SE = standard error, which was calculated after averaging the technical replicates for each umbilical cord. Live cells per gram were calculated from the live cell number for each tube divided by the weight of the tube. Theoretical yield calculation represents cell numbers achieved assuming the entire umbilical cord was processed and expanded.
