# Supplementary figures and images for: Standardizing Umbilical Cord Mesenchymal Stromal Cells for Translation to Clinical Use: Selection of GMP-Compliant Medium and a Simplified Isolation Method
Source: Stem Cells Int. 2016 Feb 4;2016:6810980. doi: 10.1155/2016/6810980 (PMC4757747; doi:10.1155/2016/6810980)

**Supplementary Table 1**

**
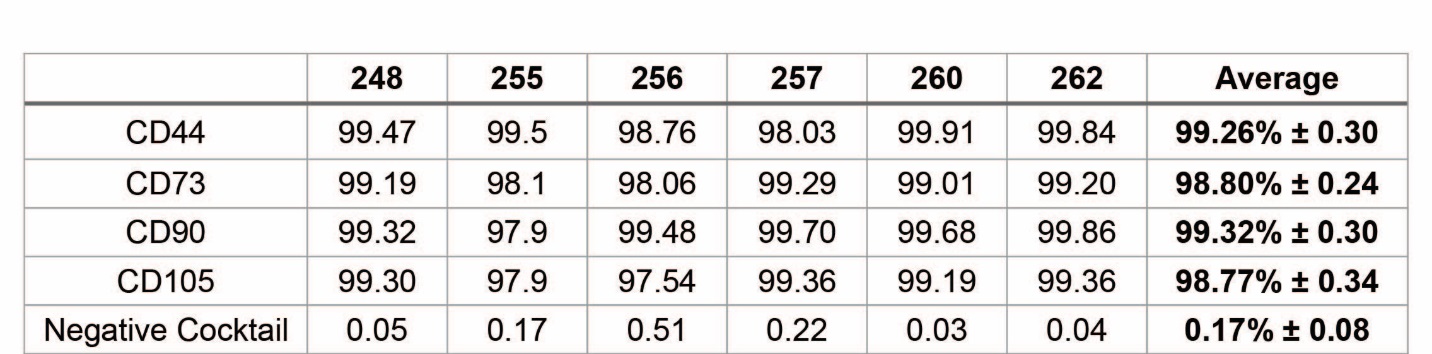
**

**Supplementary Table 2**

**
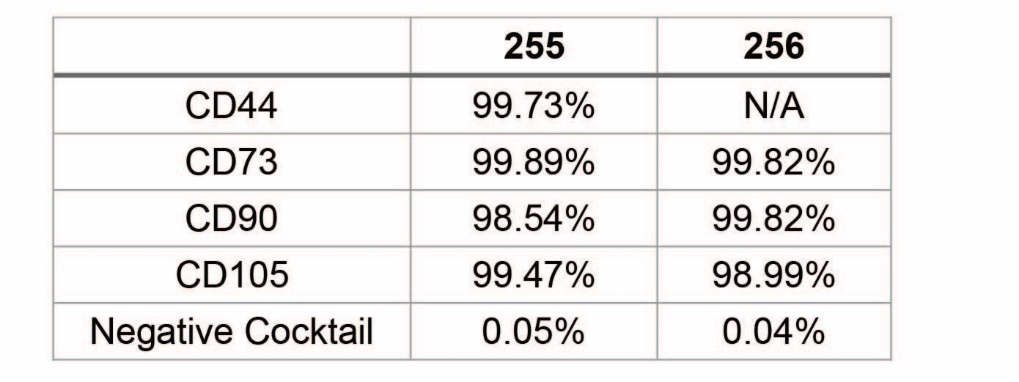
**

**Supplementary Figure 1**

**
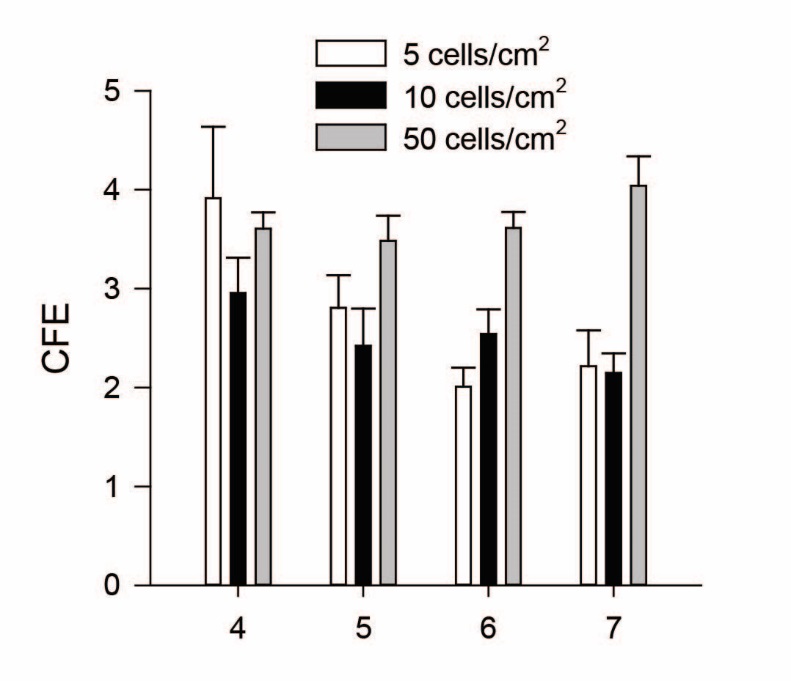
**

Supplement: Supplementary file 1 — The supplemental figures show the surface marker expression for six UC-MSC isolates and additional CFU-F information. Table 1 contains the surface marker expression data for six isolates. Note that all isolates conform to ISCT MSC surface marker criteria. In Table 2, the surface marker expression of UC-MSC isolates which have been frozen and never frozen was compared. Note that freezing did not significantly impact surface marker expression. Supplemental Figure 1 shows CFU-F expression of UC-MSCs evaluated at different times after plating. Based upon this experiment, day 6 at 5 cell per cm2 appears to yield the highest colony forming efficiency. [file 6810980.f1.docx]
